# CEO social capital and implied cost of capital: Based on the empirical evidence of Chinese family businesses

**DOI:** 10.1371/journal.pone.0316535

**Published:** 2025-03-11

**Authors:** Ya Qi, Ying Zou, Fuan Shi

**Affiliations:** School of Accounting, Capital University of Economics and Business, Beijing, China; An-Najah National University, PALESTINE, STATE OF

## Abstract

Social capital plays a crucial role in resource integration within Chinese family businesses. This research investigates the relationship between CEO social capital and the implied cost of capital, while also considering the influence of CEO type on this relationship. The empirical results based on China’s A-share family-listed companies show that CEO social capital helps to reduce the implied capital cost of family business. However, compared with non-family CEO, the effect of family CEO social capital on reducing the implied cost of capital is weaker. The mechanism analysis confirms that CEO social capital reduces the implied cost of capital through reducing corporate risk and improving information transparency. The heterogeneity analysis reveals that CEO social capital significantly reduces the implicit cost of capital only in entrepreneurial families, companies with low family control, and those without intergenerational transmission. Additionally, the effect of CEO social capital is more pronounced in fiercely competitive markets and high-tech industries. When economic policy uncertainty is high and investor legal protections are weak, CEO social capital can better exert its complementary effect on formal institutions. These findings not only provide a theoretical foundation for leveraging the informal system of social capital to strengthen family governance but also offer practical insights for addressing the classic decision of whether to choose family succession or hire professional managers.

## 1. Introduction

Over the past four decades of reform and opening up, family firms have played a vital role in driving the growth of China’s real economy. Currently, these enterprises are undergoing a critical phase of upgrading, transformation, and intergenerational succession. However, intense market competition and strict financial constraints impede their ability to pursue innovative investments and operational strategies. As a complement to formal institutions [1], social capital underscores its economic significance and serves as an effective governance mechanism for family businesses [2,3]. Particularly within the context of China’s ``relationship culture” and ``family culture,” strong social ties, a robust sense of identity, and systematic family constraints foster the generation and application of social capital.

Social capital has opened up a new research perspective for the study of family businesses. However, the existing research in the field of family businesses mainly focuses on the organizational level of family social capital, while lacking research on individual managers or non-family social capital. Especially in the practical context of limited internal resources and high external financing costs for families, it is necessary to explore the relationship between CEO social capital and implicit capital costs. Entrepreneurs can play an important role in enhancing the influence of family businesses and easing the pressure of external financing by transforming personal resources embedded in their knowledge structure and social relationships into organizational-level applications [4]. Furthermore, CEO social capital will not be transferred directly like other economic capital, but will rely on personal social networks and family kinship relationships, exhibiting a certain degree of “stickiness.” CEO social capital has become an important resource for maintaining close ties between the controlling family and external social groups, facilitating better resource allocation and decision-making.

Extensive research on the equity finance consistently indicates that the implicit capital cost reflects the expected rate of return required by investors in the stock market [5]. Stock flows and investment transactions in the capital market not only require formal systems such as securities regulations and information disclosure as guarantees, but also require the establishment of non-profit social capital. Restricted by information asymmetry, financing legality and operating uncertainty, transaction costs and risk premiums become the key factors affecting the cost of equity financing. By integrating multiple relationships beyond the family, CEO social capital establishes positive cooperative relationships and trust mechanisms with external interest groups. Consequently, CEO social capital provides an implicit soft budget constraint for family business financing and plays an active role in promoting financial transactions, reducing contract costs, and mitigating risk compensation [6,7]. This enhances the flow of information within and outside the family and reduces market surveillance and transaction costs. Formal relationship networks and trust mechanisms supplement and compensate for the inherent limitations of incomplete contracts, thereby contributing to the reduction of implicit capital cost.

During the peak period of intergenerational succession, family businesses face the decision of whether to appoint a family CEO to maintain family-style governance or to hire a non-family CEO for professional management. This dilemma has become a classic challenge in the governance of Chinese family businesses [8]. Different types of CEOs have distinct social backgrounds, knowledge resources, and cognitive foundations, which result in varying abilities to transform relationship resources from individuals to organizations. This, in turn, affects the economic activities and investment and financing decisions of family businesses. Family members serving as CEOs can help promote the continuation of social capital, such as property rights, culture, and traditions, while external professional managers bring more professional knowledge and extensive relationship networks. The social capital of different types of CEOs may exhibit varying effects on the equity financing of family businesses.

Considering China’s unique cultural context, this study aims to explore the following question: can CEO social capital synergistically govern alongside the family system to reduce transaction costs and risk compensation caused by market information risk and future uncertainty, thereby reducing the implicit capital cost of family businesses? Should a family CEO or a non-family CEO be selected to manage the company? Who can maximize the social capital effect and help family businesses obtain equity financing at a low cost? Additionally, through what specific pathways does CEO social capital influence the implicit cost of capital in family businesses? How do the family’s internal governance, industry characteristics, and macro environment affect the relationship between CEO social capital and implicit capital cost? These questions merit further exploration.

Although previous research has extensively examined social capital’s impact on corporate financing, it has not yet addressed how CEO social capital affects the cost of equity capital in family businesses. Additionally, the social capital structure and resource integration of family CEOs may differ from those of non-family CEOs. It is crucial to investigate how social capital interacts with CEO type to influence the implicit capital costs of family firms. Compared to existing literature, this article offers potential contributions: (1) capital cost research predominantly focuses on formal institutional arrangements, with limited attention to social capital as an informal institution. Few studies explore the relationship between CEO social capital and capital costs. (2) By examining the interactive effects of CEO type and social capital on implicit capital costs, this study analyzes the effectiveness of different CEO types in governing Chinese family businesses. This provides theoretical insights and practical guidance for deciding between inheriting family members or hiring professional managers. (3) Using the relationship between CEO social capital and implicit capital costs as a benchmark, this article conducts a comprehensive analysis of the active role of CEO social capital from various perspectives, such as mechanisms of action, internal family dynamics, industry characteristics, and the external macro-environment. The pathways and specific conditions of this effect further enrich the literature on family governance and social capital.

The paper is structured as follows: Section 2 presents a comprehensive literature review and outlines the research hypothesis; Section 3 provides an overview of the research design; Section 4 presents the main econometric results along with robustness tests; Section 5 offers an in-depth mechanism analysis; Section 6 conducts heterogeneity analysis, and finally, a concise summary of the entire paper is provided.

## 2. Literature review and hypothesis development

### 2.1 Social emotional wealth theory

According to socio-emotional wealth (SEW) theory, the heterogeneity of family businesses is reflected in the controlling family’s pursuit of non-economic benefits [9]. Generally, there are two main manifestations: first, constrained SEW, which focuses on maintaining family control, may even sacrifice economic interests to prevent the dilution of family control rights, resulting in a significant shortage of financial resources and investment willingness in family businesses [10]. Second, extended SEW, which focuses on ``establishing a sustainable enterprise” with a long-term development perspective, underscores the importance of aligning with the relationships between internal and external stakeholders [11]. This approach transcends the family system to attract external investors and professional talent, and building a strong stakeholder relationship network can aid in the long-term development of family businesses. In family businesses, social capital is regarded as an ``emotional domain.” A business’s ability to leverage social capital for organizational advantages depends on its capacity to maintain social relationships, integrate potential resources, and the frequency and emotional attachment in establishing social relationships [6].

### 2.2 Related research on social capital in family businesses

From the perspective of resource dependence theory, social capital is defined as ``a collection of internal and external potential resources embedded in personal or social relationships that can be transformed and applied by individuals or organizations” [12]. Social capital manifests at different levels within individuals and organizations in family businesses. At the individual level, it primarily consists of elements such as professional experience, social relationships, and professional reputation centered on the entrepreneur, including the social capital of managers, founders, or successors. At the organizational level, it permeates the enterprise through rules, systems, and shared values [6]. The dual attributes of ``family” and ``enterprise” indicate that social capital plays a crucial role in securing external information resources and equity financing for family businesses. The relationship between family governance and social capital is bidirectional [12]. Specifically, family governance necessitates the accumulation of external social capital via personal relationship networks shaped by entrepreneurs, while individual entrepreneurs simultaneously create relationship governance and integrate social capital through organizations. Additionally, social capital is cultivated through ongoing interactions that foster specific social trust and informational benefits via social relationship exchanges. These exchanges facilitate information sharing and tacit knowledge transfer, thereby enhancing the execution of loan and relational contracts [13].

### 2.3 Related research on CEO type

As the leader in corporate decision-making and external management, a CEO’s knowledge, professional experience, social relationships, and emotional preferences significantly influence strategic decisions and resource allocation [14,15]. For instance, CEOs with financial expertise can lower equity capital costs by reducing information and financial risks [16,17], while those with academic experience can decrease debt financing costs [18]. In family businesses, the CEO may be a family member or an external manager. The debate over the efficiency of family versus professional management yields mixed conclusions. Typically, non-family CEOs are seen as having superior professional skills, industry experience, and social connections, along with a greater propensity for entrepreneurial risk-taking [19,20]. Conversely, some scholars argue that external managers may prioritize short-term gains, whereas family CEOs align with long-term family interests, thereby strengthening family social capital and core competitiveness [21].

### 2.4 Research hypotheses

#### 2.4.1 CEO social capital and implicit capital cost.

CEOs are vital in the strategic decision-making of family businesses [22]. Their social capital includes management skills, reputation, industry experience, relationships, and resources. Trust and social connections provide essential resources for management activities and investment decisions [23], helping secure stakeholder support and reduce investors’ risk premiums. Therefore, the relationship between CEO social capital and implicit capital costs can be analyzed from two perspectives:

Signaling theory suggests that information asymmetry increases transaction costs and hinders efficient capital allocation. A CEO’s professional experience and resources can curb earnings management, reduce agency conflicts, and improve information disclosure. CEOs with significant social capital leverage trust to provide investors with comprehensive and accurate information. This enhances transparency, reduces transaction costs, decreases cash flow volatility, and fosters an environment where investors accept lower expected returns. Besides, Gaps in market financing contracts often lead to conflicts and opportunism. A CEO’s social capital can be viewed as an implicit contract that significantly reduces adverse selection and moral hazard [24,25]. Essentially, it serves as a protective umbrella, enhancing creditworthiness, fostering confidence within capital markets, and reducing the implicit cost of capital.

According to stakeholder theory, social capital provides reputation insurance effects and risk-sharing mechanisms [26]. Family businesses often struggle to secure resources due to a lack of professional expertise, legitimacy, and standardized institutional processes. A CEO’s personal reputation and relationship capital send positive signals to potential investors, aiding in building trust for external financing. Family businesses typically exhibit pronounced risk aversion, resulting in lower risk-taking and investment willingness. As a CEO’s social capital grows, it signifies the acquisition of essential management experience and relationship networks necessary for organizational operations. This enhances opportunities to acquire valuable resources, increases risk-taking capacity [20], and reduces investor risk compensation.

Social capital is commonly seen as an ``emotional sphere,” which is susceptible to the effects of socioemotional wealth (SEW) [6]. Constrained SEW focuses on preserving family capital, ensuring the CEO’s social capital within the family remains persistent. This social capital gradually transforms into substantial family capital through intergenerational inheritance. Conversely, extended SEW emphasizes long-term value, encouraging entrepreneurial families to hire external professional managers. This approach prompts CEO to actively maintain stakeholder relationships and build broader external social capital. It highlights the importance of fostering relationships and building trust with stakeholders, encompassing employees, customers, and suppliers alike. Concurrently, it seeks to enhance the company’s position and reputation within social networks to facilitate business success. Family enterprises thrive on a robust socioemotional wealth (SEW) environment, which effectively nurtures and sustains social capital. This SEW approach not only enhances relational governance but also facilitates resource integration. As a result, family enterprises excel at coordinating and leveraging resources within complex business environments, thereby reinforcing their competitive edge and ensuring long-term sustainability.

To sum up, the CEO serves as a bridge to foster collaboration between the family system and external social groups, helping to build substantial social capital. This enables family businesses to better integrate into the market and establish strong social relationships. By enhancing trust and support, the CEO’s social capital reduces uncertainties and risks during financing. Ultimately, investors may lower their risk premium requirements for family businesses.

H1. The richer the CEO’s social capital, the lower the equity capital cost of family businesses.

#### 2.4.2 CEO type, social capital and the implicit capital cost of family businesses.

Variations in sources, cognition, and backgrounds lead to significant differences in the impact of social capital between family and non-family CEOs. In corporate cultures that prioritize family interests, the excessive concentration of power and resources within family members can limit the participation of external talent and obstruct the company’s growth. This centralization may ultimately weaken the resources and influence that the CEO has built through social networks. The negative aspects of ``family culture,” along with an emphasis on familial interests, can diminish the positive effects of a CEO’s social capital. Non-diversifiable risks may elevate investors’ demands for risk compensation. In contrast, non-family CEOs typically have access to a broader array of social resources, enabling them to leverage external social capital to mitigate the inherent limitations of family ownership. This advantage is particularly effective in reducing the cost of equity capital.

On the one hand, family CEOs are often chosen from a limited pool of familial talent, rendering them more prone to aligning with familial objectives and economic interests. In the absence of external oversight, they may exploit pyramid structures and engage in tunneling, which can adversely affect minority shareholders and result in inefficient resource allocation and increased capital costs [27,28]. When management control is concentrated among a small group of family members, nepotism and conflicts of interest may persist [29,30], potentially resulting in management risks and undermining the legitimacy of external resource acquisition. This is because these members may be reluctant to relinquish control or change the existing distribution of benefits. Additionally, the appointment of family members may encounter resistance from non-family shareholders, increasing uncertainty regarding future cash flows for external investors.

On the other hand, external professional managers typically possess a substantial reservoir of expertise and managerial prowess. They effectively mitigate the inclination of major shareholders to conspire, thereby reinforcing the legitimacy of external equity financing and reducing the risk premiums sought by market participants [31]. Unlike family CEOs, who may rely on tight-knit internal relationships, non-family executives leverage their professional and social competencies to navigate external stakeholder networks more effectively. This enables them to surmount political and economic challenges with greater ease and finesse, thereby bolstering the market competitiveness of family businesses. As non-kin elements within the family governance framework, these external managers collaborate with the established governance mechanisms to elevate investor confidence and trust [8], while concurrently diminishing credit risk and uncertainty. This synergistic effect enables the company to secure equity capital at a reduced cost of financing. Consequently, Hypothesis 2 is proposed.

H2. CEO type negatively moderates the inverse relationship between social capital and the implied cost of capital.

## 3. Research design

### 3.1 Sample and data

This article selects A-share family companies in China’s Shanghai and Shenzhen stock markets from 2008 to 2021. Relevant data involving family information and financial indicators are mainly collected based on the CSMAR databases. CEO social capital data is mainly based on executive resume information in the CSMAR database, and is manually compiled from websites such as Baidu, Google, and Oriental Fortune Network.

Given the lack of consensus in the academic literature regarding the definition of family businesses [32], this paper adopts a determination process informed by existing research. The selection criteria include: (1) the ultimate controller must be identified as a natural person or a family; (2) the ultimate controller must be the largest shareholder, whether directly or indirectly, of the listed company; (3) the actual controller of the company must belong to a specific family, with relatives holding positions as shareholders, directors, supervisors, or senior management within the listed company or its controlling shareholder entity.

In line with this definition, an initial screening of the “actual controller type” field was conducted to include only natural persons or families, excluding those companies operating in the financial and insurance sectors, as well as those designated as ST or * ST to reduce the influence of outliers. Continuous variables were adjusted by winsorizing at the 1st and 99th percentiles to ensure a more balanced dataset. Following these procedures, an unbalanced panel dataset consisting of 11,812 observations was compiled for the purposes of this study. The data analysis and empirical testing were performed using Stata 17 software.

### 3.2 Model setting

To investigate the benchmark relationship between CEO social capital and the implicit cost of capital, this study employs a fixed effects model. To address potential endogeneity issues stemming from time-invariant omitted variables, the analysis primarily utilizes a two-way fixed effects model that accounts for both company-specific and year-specific fixed effects.

The model is specified as follows


ICC=α1SC+α2∑Controlsit+αt∑Year +Ci +εit (1)

ICC=β1SC+β2Ceotype+β3SC×Ceotype+β4∑Controlsit+βt∑Year+Ci +εit (2)

Among them, controls represents the set of all control variables, *α*_*1*_*~α*_*2*_, *β*_*1*_*~β*_*4*_ is the regression coefficient, *C*_*i*_ is the fixed effect of individual companies, *α*_*t*_, *β*_*t*_ is a fixed time effect, *ε*_*it*_ is the residual term.

### 3.3 Variable definition

#### 3.3.1 Independent variable-CEO social capital.

Building on the research of Sanchez-Ruiz et al. (2019) [33], this study constructs a measurement approach for CEO social capital within the context of family businesses. The measurement is based on eight dimensions of internal and external social capital, and a principal component analysis is conducted to derive a composite index (SC) of CEO social capital for family businesses. The specific methodology for calculating and assigning values is presented in Table 1.

**Table 1:** Measurement and valuation methods of CEO social capital.

Dimension	Aspect	Valuation
Internal social capital	Personal Reputation	National honors or titles = 3, Provincial level = 2, Municipal level and below = 1
	Knowledge Structure	Doctorate or graduate degree = 3, Undergraduate degree = 2, College degree or below = 1
	Serving Year	Number of years as CEO of the family business
	Career background	Management, marketing, human resources = 3, Legal and financial affairs = 2, Production and R&D = 1
External social capital	Overseas relations	Working overseas = 3, Studying abroad = 2, Other international experience = 1
	Financial relations	Financial regulatory agencies = 3, Policy or commercial banks = 2, Insurance and securities = 1
	Political relations	National People’s Congress or CPPCC member = 3, Local government experience = 2, Research or academic experience = 1
	Business relations	Chamber of commerce president or vice-president = 3, Chamber of commerce member = 2, Part-time role in other enterprises = 1

#### 3.3.2 Dependent variable-implied cost of capital (ICC).

The measurement of the cost of equity capital typically follows two main approaches: ex ante and ex post. The implied cost of capital is predominantly estimated prospectively. It primarily reflects market investors’ expectations for future investment returns and effectively mitigates the estimation errors associated with the standard CAPM model. Consequently, it enriches the informational content of the cost of capital and enhances the precision of its measurement. Forecasted surplus serves as a crucial basis for ex ante capital cost estimation, with its sources primarily including analyst forecasts and cross-sectional rolling regression forecasts. Drawing on relevant research, this study employs analysts’ forecasted earnings, the Hvz model, and the modified RI model as core data for capital cost estimation, along with the Gordon [34], PEG [35], GLS [36], and OJ models [37]. The mean value of the four types of capital cost estimates across different forecast earnings dimensions is used as the proxy variable (ICC) for the implied cost of capital. The specific estimation method and key parameters are detailed in Table 2.

**Table 2:** Implicit capital cost estimation model and main parameters.

Method	Model	Specific estimation method	Main parameter description
Implied cost of capital	Gordon	${\text{r}}_{\text{e}}=\frac{{\text{dps}}_{1}}{{\text{P}}_{0}}+\text{g}$.	(1) P_0_: Closing price at the end of the previous year; (2) K_0_:Actual dividend payout rate at the end of the previous year; (3) bps: Net assets per share at the end of the previous year; (4) eps (earnings per share): Four types of data sources ① Actual data; ② Analyst forecast data; ③ HVZ model cross-sectional regression predictions; ④ MRI model cross-sectional regression predictions (5) dps (dividends per share): ${\text{dps}}_{1}={\text{eps}}_{1}\times {\text{K}}_{0}$ (6) γ-1, g: 10-year Treasury bond yield (7) roe _t_ = eps _t_/ bps _t-1_
	PEG	$r_{e}=\sqrt{\frac{{\text{eps}}_{\text{t}+2}-{\text{eps}}_{\text{t}+1}}{{\text{P}}_{\text{t}}}}$	
	GLS	${\begin{array}{c} P \end{array}}_{0}={\text{bps}}_{0}+\overset{3}{\underset{\text{t}=1}{\sum }}\frac{（{\text{roe}}_{\text{t}}-{\text{r}}_{\text{e}}）}{（1+{\text{r}}_{\text{e}}{）}^{\text{t}}}{\text{bps}}_{\text{t}-1}+\overset{11}{\underset{\text{t}=4}{\sum }}\frac{（{\text{roe}}_{\text{t}}-{\text{r}}_{\text{e}}）}{（1+{\text{r}}_{\text{e}}{）}^{\text{t}}}{\text{bps}}_{\text{t}-1}+\frac{（{\text{roe}}_{12}-{\text{r}}_{\text{e}}）}{{\text{r}}_{\text{e}}（1+{\text{r}}_{\text{e}}{）}^{11}}{\text{bps}}_{11}$	
	OJ	${\text{r}}_{\text{e}}=\text{A}+\sqrt{{\text{A}}^{2}+\frac{{\text{eps}}_{\text{t}+1}}{{\text{P}}_{\text{t}}}\times \left(\frac{{\text{eps}}_{\text{t}+2}-{\text{eps}}_{\text{t}+1}}{{\text{eps}}_{\text{t}+1}}-\left(\text{γ}-1\right)\right)}$ $\text{A}=\frac{1}{2}（\text{γ}-1+\frac{{\text{dps}}_{\text{t}+1}}{{\text{P}}_{\text{t}}}）$	

#### 3.3.3 Moderating variable-CEO type.

Drawing on the research of Mullins et al. (2016) [38], CEO type is categorized into two groups: family CEOs and external non-family CEOs. A family CEO is defined as a manager who is a member of the founder’s family, assigned a value of ``1.” Conversely, non-family CEOs are individuals unrelated to the company owner, typically professional managers from external backgrounds, and are assigned a value of ``0.”

#### 3.3.4 Control variables.

Drawing from existing literature on capital costs and family businesses, we include several control variables: company size (Size), business age (Age), capital structure (Lev), corporate growth (Growth), dual-job integration (Duel), profitability (Roa), family ownership ratio (Famown), and equity balance (Balance). These variables help ensure that the regression results are both objective and accurate. Additionally, this study controls for sample year and firm-specific fixed effects. Definitions of the specific variables are provided in Table 3.

**Table 3:** Variable definition table.

Type	Name	Definition and measures
Dependent variable	ICC	The average cost of capital of Gordon, GLS, PEG and OJ models.
Independent variable	SC	The comprehensive score of CEO social capital obtained by principal component analysis in this paper.
Mediation variable	Ceotype	Assigned a value of 1 if the CEO is a family member, and 0 if the CEO is an externally hired manager.
Control variable	Age	The total number of years of operations.
	Size	The natural logarithm of total assets.
	Growth	The growth rate of main operating revenue.
	Lev	The ratio of total liabilities to total assets.
	Duel	Assigned a value of 1 if the chairman and general manager hold the position concurrently, and 0 otherwise.
	Famown	The ownership (voting rights) held by the actual controlling family.
	Roa	The ratio of corporate profits to average corporate assets.
	Balance	The shareholding ratio of the top five shareholders to the largest shareholder.
	Year	Time dummy variables representing the years 2008 to 2021.

## 4. Empirical analysis

### 4.1 Descriptive statistics

The descriptive statistics for the main variables are presented in Table 4. The mean implicit capital cost (ICC) for family enterprises in the full sample is 8.854%, with a standard deviation of 3.587, indicating significant variability in equity financing costs among Chinese family businesses. After conducting principal component analysis, the mean score for social capital (SC) is 2.031, with a maximum of 3.686 and a minimum of -0.0397, reflecting substantial differences in the richness of CEO social capital across various family companies. Notably, the mean of the CEO type variable (Ceotype) is 0.608, suggesting that more than half of the family businesses appoint family members as CEOs. This finding establishes a foundation for further exploration of the relationship between CEO social capital and implicit capital costs across different CEO types. The mean family ownership ratio (Famown) is 38.22, with a standard deviation of 16.57, indicating a considerable disparity in family ownership proportions among the firms.

**Table 4:** Sample statistics.

Variable	N	Mean	Sd	Min	P50	Max
ICC	118 12	8.854	3.587	3.131	8.233	26.33
SC	118 12	2.031	0.675	-0.0397	2.010	3.686
Ceotype	118 12	0.608	0.488	0	1	1
Age	118 12	15.89	5.815	4	16	31
Size	118 12	21.80	1.062	19.13	21.69	24.87
Growth	118 12	0.334	0.806	-0.759	0.138	6.551
Roa	118 12	0.0602	0.0423	-0.395	0.0529	0.227
Lev	118 12	0.362	0.184	0.0447	0.349	0.987
Famown	118 12	38.22	16.57	6.121	36.72	78.43
Balance	118 12	0.847	0.639	0.0147	0.686	4
Duel	118 12	0.412	0.492	0	0	1

### 4.2 Related analysis

Table 5 presents the correlations among the core variables. The correlation coefficient between CEO social capital (SC) and implicit capital cost (ICC) is -0.104 (*p <  .01*), indicating a statistically significant negative relationship at the .01 significance level. This finding supports Hypothesis 1, providing basic evidence for the assertion that higher CEO social capital is associated with lower implicit capital costs.The coefficient between CEO type (Ceotype) and ICC is 0.0090 (*p >  .1*). Although this positive correlation is not statistically significant, it suggests that appointing family members as CEOs may not benefit family firms in terms of reducing capital costs.

**Table 5:** Correlation analysis of main variables.

Variable	ICC	SC	Ceotype	Lev	Famown	Balance
ICC	1.0000					
SC	-0.104***	1.0000				
Ceotype	0.0090	0.181***	1.0000			
Lev	0.103***	-0.018*	-0.106***	1.0000		
Famown	0.057***	-0.0090	0.182***	-0.121***	1.0000	
Balance	0.0120	-0.017*	0.047***	-0.089***	-0.144***	1.0000

Note: * p <  .1, ** p <  .05, *** p <  .01

Additionally, the correlation coefficient between family ownership (Famown) and ICC is 0.057 (*p <  .01*), indicating that a higher degree of family control does not contribute to a reduction in corporate equity financing costs. The correlation between Famown and SC is -0.0090 (*p >  .1*); while not significant, this suggests that under conditions of high family ownership, CEO social capital may be relatively scarce. This observation implies that excessive family control can negatively impact the relationship between CEO social capital and capital costs, providing a foundation for the subsequent mechanism analysis and further discussions in this paper.

### 4.3 Baseline regression results

To validate the suitability of the fixed-effects model, this study employs both random-effects and fixed-effects regressions on unbalanced panel data, followed by a Hausman test. The p-value of less than .01 indicates that the fixed-effects model is statistically more suitable. Additionally, incorporating dual fixed effects for time and individual can help mitigate the issue of omitted variables that remain constant over time at the individual level. After accounting for individual and time-specific fixed effects, we introduce further control variables for the panel fixed-effects regression.

The benchmark regression results are shown in Table 6. Column (3) presents the regression findings without control variables, showing a regression coefficient of -0.3364 for CEO social capital (SC). At the 1% significance level, this indicates a significant negative correlation between CEO social capital and the implicit capital cost of family enterprises. With the introduction of control variables, the regression coefficient for SC in column (4) increases to -0.3796 (t =  -3.7859, p <  .01). Correspondingly, the model’s goodness of fit improves from 0.1256 to 0.2927, suggesting enhanced model fit following the addition of these control variables.

**Table 6:** CEO social capital and implicit capital cost.

Variables	Random effects		Fixed effects	
	(1) ICC	(2) ICC	(3) ICC	(4) ICC
SC	-0.5283 ^***^ (-10.2713)	-0.3092 ^***^ (-4.4406)	-0.3364 ^***^ (-2.9379)	-0.3796 ^***^ (-3.7859)
Age		0.0196 ^**^ (2.3602)		0.3875 ^*^ (1.9207)
Size		-0.5499 ^***^ (-9.4280)		-1.0315 ^***^ (-9.1056)
Growth		0.2484 ^***^ (4.9903)		0.2813 ^***^ (4.9473)
Roa		40.9828 ^***^ (33.3924)		43.7264 ^***^ (29.5295)
Lev		5.7888 ^***^ (18.4709)		4.9293 ^***^ (11.1878)
Famown		-0.0008 (-0.3118)		-0.0077 (-1.3404)
Balance		0.0845 (1.2749)		0.1952 (1.2889)
Duel		0.2195 ^***^ (2.7655)		0.4036 ^***^ (3.4077)
Constant	10.0191 ^***^ (90.2084)	10.5460 ^***^ (12.9050)	9.1226 ^***^ (25.7655)	22.3358 ^***^ (7.7526)
Time fixed effects	Yes	Yes	Yes	Yes
Firm fixed effects	No	No	Yes	Yes
N	11812	11812	11812	11812
adj. R ^2^	0.0109	0.2909	0.1256	0.2927

*Note*: In parentheses are the t values of the corresponding coefficients, reflecting the robust standard errors adjusted for heteroskedasticity, ^*^ *p* <  0.1, ^**^ *p* <  0.05, ^***^ *p* <  0.01 (same as the table below).

The regression results clearly indicate that greater CEO social capital is associated with lower implicit capital costs for family businesses. These businesses are particularly motivated to preserve non-economic benefits, such as social and emotional wealth, which positions them to navigate financing challenges using the informal mechanisms provided by the CEO’s social capital. Specifically, by increasing the participation of family business stocks in the capital market, these strategies help reduce market oversight and transaction costs, thereby enhancing the efficiency of resource allocation.

Conversely, for family businesses facing significant financial constraints, both internal and external social capital play crucial roles in reputation protection and risk-taking. By expanding financing avenues and reducing information asymmetry, these resources enhance market reputation, resulting in greater recognition from stakeholders and investors. This increased recognition subsequently decreases the risk premiums associated with informational risks and uncertainties, ultimately lowering the implicit capital costs for family businesses. Therefore, the findings of this study support Hypothesis 1.

### 4.4 Analysis of dynamic adjustment of CEO type

Given the significant differences in social capital structures and the extent of resource transformation among CEOs from various backgrounds, the impact of CEO type on the implied cost of capital may differ considerably. This paper distinguishes between family CEOs and externally hired non-family CEOs, examining the interactive effects of CEO type and social capital on the implied cost of capital in family businesses. The specific effects are presented in Table 7.

**Table 7:** The moderating effect of CEO type.

Variables	(1) ICC	(2) ICC
SC	-0.6246^***^ (-4.7316)	-0.4896^***^ (-4.3345)
Ceotype	-0.3423 (-1.3045)	-0.4780^**^ (-2.0925)
SC × Ceotype	0.3548^***^ (2.9519)	0.1749^*^ (1.7413)
Age		0.3880^*^ (1.9245)
Size		-1.0281^***^ (-9.0813)
Growth		0.2786^***^ (4.8991)
Roa		43.7010^***^ (29.5293)
Lev		4.9312^***^ (11.1901)
Famown		-0.0072 (-1.2505)
Balance		0.1977 (1.3069)
Duel		0.5041^***^ (3.3451)
Constant	9.4564^***^ (25.3170)	22.4955^***^ (7.8086)
Time fixed effects	Yes	Yes
Firm fixed effects	Yes	Yes
N	11,812	11,812
adj. R ^2^	0.1271	0.2929

Column (1) presents the univariate regression results, which include CEO type and the interaction term. The coefficient for the SC ×  CEO type interaction term is 0.3548 (t = 2.9519), with p <  0.01. This indicates a significant interaction effect. Notably, the sign of the SC coefficient is opposite to that of the interaction term coefficient.

Column (2) highlights the moderating effect of CEO type after incorporating a series of control variables. The independent variable SC coefficient is -0.4896 (t =  -4.896), with p <  0.01, while the coefficient for the interaction term is 0.1749 (t =  1.7413), with p <  0.1. These results suggest that CEO type weakens the negative correlation between social capital and the implied cost of capital. Specifically, family CEOs, compared to professional non-family CEOs, often lack legitimacy and professional management expertise, and may even harbor collusive motives that align with the interests of the controlling family. Consequently, the limited social capital of family CEOs makes it difficult for them to effectively protect reputation and share information risks. The concentration of family interests can lead to operational risks that diminish the positive influence of a CEO’s social capital.

**Fig 1:**
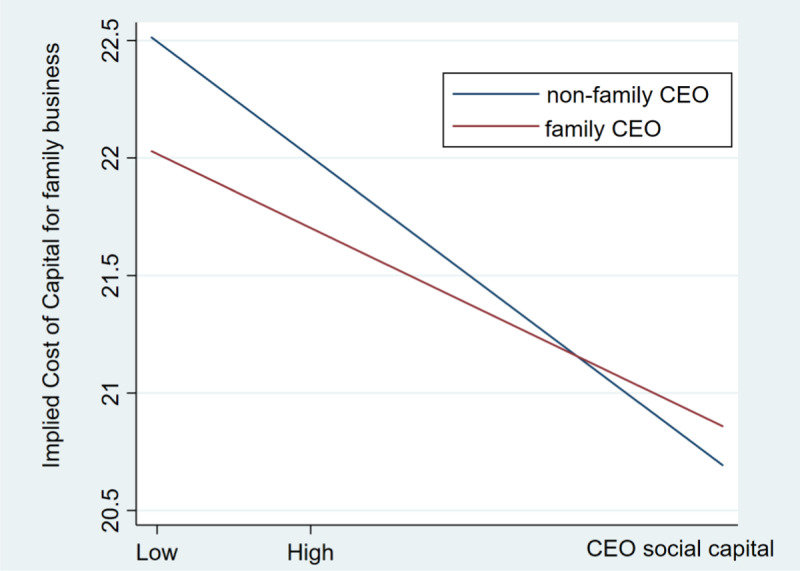
Moderating effect diagram of CEO type.

Furthermore, we employ Stata software to depict the moderating effect of CEO type on the primary relationship investigated in this study, as showed in Fig 1. Despite both curves showing a downward trend, the slope for non-family CEOs is notably steeper compared to that of family CEOs. This emphasizes that non-family CEOs with elevated social capital exhibit greater proficiency in decreasing the implied cost of capital. In contrast, when CEOs are family members, the mitigating influence of social capital on the implied cost of capital is attenuated. This finding further supports the validity of Hypothesis 2.

### 4.5 Robustness test

#### 4.5.1 Replacement of the explained variable.

In previous analyses, the average value obtained from various estimation methods was used as the measurement index for the implied cost of capital. However, given that the implied cost of capital may exhibit outliers or estimation deviations across different estimation methods, the averaging process could introduce estimation errors, potentially impacting the empirical regression results.

To address this issue, the classical method for estimating ex ante equity capital costs is employed, specifically the Gordon Growth Model (GGM). This approach estimates the implied capital cost separately, and the explained variables are replaced to re-verify the earlier conclusions. The test results are summarized in Table 8. Column (1) presents the main effect, where the social capital (SC) coefficient is -0.3179 (*p <  0.01*).Column (2) illustrates the moderation effect, with the coefficient of the interaction term being 0.2835 (*p <  0.05*). This coefficient has an opposite sign to that of the SC coefficient, thereby reinforcing our previous conclusions.

**Table 8:** Robustness check: replacement and lagged variables.

Variables	(1) ICC_ggm	(2) ICC_ggm	(3) L.ICC	(4) L.ICC	(5) ICC	(6) ICC	(7) ICC	(8) ICC
SC	-0.3179 ^***^ (-2.7145)	-0.5047 ^***^ (-3.6595)	-0.2416 ^**^ (-2.1518)	-0.3303 ^**^ (-2.4271)	-0.0448 ^***^ (-4.1652)	-0.0363 ^**^ (-2.4394)		
Ceotype		-0.4990 ^*^ (-1.8728)		-0.1095 (-0.3884)		0.0006 (0.0022)		-0.3569 (-0.9999)
Sc×Ceotype		0.2835 ^**^ (2.3748)		0.1306 ^*^ (1.0382)		-0.0121 ^*^ (-0.6696)		
L.SC							-0.2708 ^***^ (-2.7361)	-0.3397 ^**^ (-2.3587)
L.SC×ceotype								0.1133 ^*^ (0.6850)
Age	0.0609 (0.3595)	0.0667 (0.3934)	0.5629 ^**^ (2.3830)	0.5701 ^**^ (2.4230)	0.3017 ^*^ (1.8471)	0.2991 ^*^ (1.8255)	0.0631 (0.3443)	0.0615 (0.3334)
Size	0.0241 (0.1802)	0.0286 (0.2146)	0.3280 ^***^ (2.6435)	0.3295 ^***^ (2.6552)	-0.9771 ^***^ (-9.5939)	-0.9769 ^***^ (-9.5935)	-0.7005 ^***^ (-7.4173)	-0.6991 ^***^ (-7.4099)
Growth	0.3422 ^***^ (4.2668)	0.3397 ^***^ (4.2383)	0.0445 (0.6646)	0.0444 (0.6603)	0.2812 ^***^ (5.1776)	0.2805 ^***^ (5.1653)	0.2231 ^***^ (4.1765)	0.2224 ^***^ (4.1723)
Roa	161.7633 ^***^ (72.1150)	161.6938 ^***^ (72.1381)	34.7650 ^***^ (22.9473)	34.7252 ^***^ (22.9534)	43.5293 ^***^ (34.1118)	43.5483 ^***^ (34.1076)	40.3069 ^***^ (31.8203)	40.3106 ^***^ (31.8811)
Lev	16.6008 ^***^ (30.4216)	16.6107 ^***^ (30.4492)	-0.5256 (-1.0682)	-0.5141 (-1.0442)	4.7920 ^***^ (12.0455)	4.7895 ^***^ (12.0272)	6.1290 ^***^ (16.2486)	6.1178 ^***^ (16.2455)
Famown	-0.0028 (-0.4183)	-0.0029 (-0.4379)	0.0155 ^**^ (2.3525)	0.0150 ^**^ (2.2587)	-0.0061 (-1.1305)	-0.0056 (-1.0468)	-0.0132 ^**^ (-2.3670)	-0.0128 ^**^ (-2.3026)
Balance	-0.0893 (-0.5910)	-0.0893 (-0.5922)	0.1277 (0.6629)	0.1272 (0.6630)	0.1817 (1.2674)	0.1821 (1.2677)	-0.2441 ^*^ (-1.7647)	-0.2417 ^*^ (-1.7390)
Duel	0.0773 (0.5930)	0.0472 (0.2909)	0.3638 ^**^ (2.4516)	0.2652 (1.4140)	0.3888 ^***^ (3.4507)	0.4930 ^***^ (3.4285)	0.0573 (0.5592)	0.1386 (1.0472)
_cons	-7.8065 ^**^ (-2.5339)	-7.6070 ^**^ (-2.4734)	-6.2023 ^*^ (-1.8366)	-6.1632 ^*^ (-1.8322)	21.6486 ^***^ (8.6302)	21.6006 ^***^ (8.5968)	20.2124 ^***^ (7.5421)	20.3673 ^***^ (7.6108)
fixed time	Yes	Yes	Yes	Yes	Yes	Yes	Yes	Yes
Firm fixed	Yes	Yes	Yes	Yes	Yes	Yes	Yes	Yes
N	11812	11812	9138	9138	11812	11812	9138	9138
adj. R ^2^	0.7241	0.7242	0.2341	0.2341	0.3050	0.3050	0.4420	0.4421

#### 4.5.2 One-period lagged explained variable.

To account for the potential delay in the impact of CEO social capital on family businesses, it is important to recognize that the social capital accumulated by a CEO may take time to be effectively translated into organizational benefits, ultimately influencing the capital costs of the family business. Therefore, this paper conducts a robustness test by implementing a one-period lag for the dependent variable’s implied cost of capital.

The test results are presented in columns (3) and (4) of Table 8. At the 0.05 significance level, the main effect coefficient for social capital (SC) remains negative, while the interaction coefficient is significant at the 0.1 significance level and has an opposite sign to the SC coefficient. These findings indicate that the previous conclusions are robust, reinforcing the validity of the observed relationships.

#### 4.5.3 Measurement method of replacing explanatory variables.

In the previous benchmark test, CEO social capital was measured using a comprehensive score obtained through principal component analysis (PCA). However, this approach may introduce a standard selection bias. To address this concern, this article employs a direct summation of CEO social capital indicators for robustness testing. The results, as shown in columns (5) and (6) of Table 8, indicate that the SC coefficient is -0.0448 (p <  0.01), while the interaction term is -0.0121 (p <  0.1). These findings confirm that the previous conclusions remain robust.

#### 4.5.4 The explanatory variables lagged by one period.

There may be concerns regarding reverse causality and sample self-selection between CEO social capital and the cost of capital in family businesses. To address potential endogeneity issues, the core variable, SC, is lagged by one period for robustness testing.

The results are presented in columns (7) and (8) of Table 8, where the lagged SC coefficient is -0.2708 (p <  0.01), and the coefficient for the interaction term with lagged SC is 0.1133 (p <  0.1). These findings further confirm the robustness of the previous conclusions.

#### 4.5.5. Split-sample regression.

The findings discussed above confirm the moderating effect of CEO type on the fundamental relationship between CEO social capital (SC) and the implicit cost of capital (ICC). To further validate these conclusions, a robustness test will be carried out by splitting the sample into sub-samples based on CEO type: family CEO and non-family CEO.

Results are presented in Table 9. In column (1), which represents the sample of family CEOs, the coefficient of SC is -0.1562 (*p >  0.1*), indicating a negative correlation between CEO social capital and the implicit cost of capital, although this relationship is not statistically significant. Conversely, in column (2), which presents the regression results for non-family CEOs, the coefficient of SC is -0.5027 (*p <  0.1*). This indicates a stronger negative correlation between SC and ICC for non-family CEOs, and the result is significant at the 0.01 level. These findings confirm that social capital among non-family CEOs is more effective in reducing the implicit cost of capital, thus supporting the reasoning and conclusions drawn in the hypothesis.

**Table 9:** Robustness check: changing the regression sample.

Variables	Split-sample regression		Redefine the sample	
	(1) IRR	(2) IRR	(3) IRR	(4) IRR
SC	-0.1562 (-1.0937)	-0.5027 ^***^ (-2.9504)	-0.3674 ^***^ (-3.3537)	-0.4656 ^***^ (-3.7388)
Ceotype				-0.3483 (-1.4185)
SC×Ceotype				0.1489 ^*^ (1.4012)
Age	0.4135 ^*^ (1.7457)	0.1624 (1.0092)	0.2699 (1.4242)	0.2714 (1.4332)
Size	-1.0747 ^***^ (-7.0977)	-1.0083 ^***^ (-6.1492)	-0.9572 ^***^ (-7.9302)	-0.9554 ^***^ (-7.9156)
Growth	0.3565 ^***^ (4.0219)	0.1928 ^***^ (2.8821)	0.3368 ^***^ (5.5908)	0.3350 ^***^ (5.5639)
Roa	43.2493 ^***^ (24.6337)	42.0772 ^***^ (20.0539)	43.1200 ^***^ (31.3433)	43.0845 ^***^ (31.2977)
Lev	4.6840 ^***^ (8.4274)	5.6408 ^***^ (9.1532)	4.7363 ^***^ (10.6137)	4.7331 ^***^ (10.6036)
Famown	-0.0068 (-0.9381)	-0.0050 (-0.5312)	-0.0137 ^**^ (-2.0050)	-0.0134 ^**^ (-1.9667)
Balance	0.3381 ^*^ (1.7004)	0.2006 (1.0696)	0.2461 (1.2442)	0.2482 (1.2558)
Duel	0.5069 ^***^ (2.7060)	2.6574 ^***^ (3.0352)	0.4025 ^***^ (3.1519)	0.4467 ^***^ (2.8274)
Constant term	22.9713 ^***^ (6.2774)	23.6852 ^***^ (6.3539)	22.5429 ^***^ (7.7627)	22.6902 ^***^ (7.7995)
time fixed effects	Yes	Yes	Yes	Yes
firm fixed effects	Yes	Yes	Yes	Yes
N	7182	4630	10104	10104
adj. R ^2^	0.2797	0.3444	0.2862	0.2862

#### 4.5.6 Redefining the sample.

The lack of uniform standards for defining family businesses in existing research may lead to biased results. To address this issue, a threshold of 20% family ownership is adopted as the criterion for redefining the family business sample. This redefinition aims to verify the validity of the previously drawn conclusions.

As displayed in columns (3) and (4) of Table 9, the SC coefficient remains significantly negative at the .01 significance level. This indicates that the moderating effect persists even after redefining the sample, thereby reinforcing the robustness of the aforementioned conclusions.

## 5. Further discussion

### 5.1 Mechanism analysis

This study has established that CEO social capital significantly reduces the implicit cost of capital. In this section, we will delve deeper into the specific mechanisms behind this relationship, focusing on two key factors: enterprise risk and information transparency. To further examine these mechanisms, we will build upon the previously established model (1) and construct a mediation mechanism model. This model will allow us to explore how enterprise risk and information transparency mediate the effect of CEO social capital on the implicit cost of capital, thereby providing a more comprehensive understanding of the dynamics at play.


Risk =γ1SC+γ2∑Controls it +γt ∑Year +Ci +ε it (3)

ICC=θ1SC +θ2Risk +θ3∑Controls it +θt ∑Year +Ci +εit (4)

Trans =λ1SC+λ2∑Controls it +λt ∑Year +Ci +εit (5)

ICC=n1SC +η2Trans +η3∑Controls it +ηt ∑Year +Ci +εit (6)

Among the variables analyzed, risk serves as the proxy for enterprise risk. We employ the three-period rolling standard deviation of industry-adjusted asset returns as the measure for enterprise risk. A higher value of Risk indicates greater enterprise risk exposure. The proxy for information transparency (Trans) is constructed using various indicators, including corporate earnings information. A higher value of Trans signifies greater transparency in the information disclosed by the enterprise. According to the mediation test procedures, a significant mediation effect of *Risk* is indicated when the coefficients *α*_*1*_, *γ*_*1*_, *θ*_*2*_ are significant. Furthermore, if the coefficient *θ*_1_ is also significant, this would indicate a partial mediation effect. In a similar vein, a partial mediation effect regarding information transparency is indicated when all coefficients *α*_1_, *λ*_1_, *η*_1_, *η*_2_ are significant.

#### 5.1.1 Risk-taking.

Based on the existing literature, it is observed that family businesses often exhibit a relatively low level of risk-taking behavior due to the impact of constrained Socioemotional Wealth (SEW) [20]. This excessive concentration on the controlling interests of the family exacerbates operational risks, resulting in diminished legitimacy regarding external financing for these businesses. However, when the CEO possesses substantial social capital, their professional management experience and knowledge can enhance corporate governance structures. Moreover, the CEO’s personal social network, which is frequently integrated within the family business, facilitates resource integration. This dynamic contributes to reducing the company’s overall risk level, improves the perception of external stakeholders, and ultimately encourages market investors to lower their risk premiums.

To empirically investigate the pathway ``CEO social capital →  enterprise risk level →  implicit cost of capital,” this study utilizes models (1), (3), and (4). The results of the mediation mechanism test are summarized in Table 10. As shown in Column (2), the regression coefficient for the relationship between CEO social capital (SC) and the intermediary variable risk is -0.0050, which is significant at the 0.05 level. This finding suggests that CEO social capital effectively reduces the company’s risk level.

**Table 10:** Examination of the mediating mechanism.

Variables	Risk-taking			Information transparency		
	(1) IC _	(2) Risk	(3) IC _	(4) IC _	(5) T rans	(6) IC _
SC	-0.3737 ^***^ (-4.3973)	-0.0050 ^**^ (-2.4095)	-0.3462^***^ (-4.0496)	-0.3622 ^***^ (-4.2007)	0.0261 ^***^ (7.3605)	-0.2781 ^***^ (-3.2451)
Risk			5.5403 ^***^ (13.3758)			
Trans						-3.2191 ^***^ (-13.2792)
_cons	22.3219 ^***^ (12.0882)	-0.1950 ^***^ (-4.4096)	23.4023 ^***^ (12.7778)	21.6932 ^***^ (11.6184)	-1.3278 ^***^ (-17.2764)	17.4189 ^***^ (9.2725)
Control variables	Yes	Yes	Yes	Yes	Yes	Yes
Time fixed effects	Yes	Yes	Yes	Yes	Yes	Yes
Firm fixed effects	Yes	Yes	Yes	Yes	Yes	Yes
N	11802	11802	11802	11690	11690	11690
adj. *R*^2^	0.1542	0.0130	0.1798	0.1410	-0.0147	0.1560

Furthermore, Columns (1) and (3) show that after incorporating the intermediary variable risk into the benchmark regression model, the coefficient for SC becomes -0.3462 (P <  0.01), while the coefficient for risk is 5.5403 (P <  0.01). These results indicate a significant partial mediation effect, confirming that CEO social capital can lower the company’s risk level and, consequently, reduce the implied cost of capital for family businesses.

#### 5.1.2 Information transparency.

Under the family governance structure of ``one-share dominance,” information asymmetry significantly affects financing costs. A CEO’s abundant social capital enhances their professional experience and access to informational resources, which helps reduce earnings management practices and improve the quality of information disclosure. Social capital allows external investors to obtain more comprehensive and accurate information, increasing the transparency of family business operations. It also lowers transaction costs and risk premiums linked to information asymmetry. Moreover, the legitimacy of family business financing often faces scrutiny from market participants. CEO social capital can address various adverse selection and moral hazard issues through trust mechanisms and social networks, thereby reinforcing the legitimacy of equity financing for family businesses. The benefits of social capital motivate stakeholders to gain a deeper understanding of the enterprise’s future prospects and operational conditions, leading to improved corporate transparency and ultimately lowering the implied cost of capital for family businesses.

This paper employs models (1), (5), and (6) to test the validity of the pathway “CEO social capital → information transparency → implied cost of capital.” The results of this analysis are presented in Table 10. Column (5) shows that the regression coefficient between social capital (SC) and transparency (Trans) is 0.0261 (p <  0.01), indicating that CEO social capital significantly enhances the transparency of family business information. Furthermore, in Column (6), the inclusion of the mediating variable Trans confirms that the coefficients for SC and Trans remain significantly negative at the 0.01 level, demonstrating that *Trans* plays a partial mediating role.

### 5.2 Group discussion

The analysis has confirmed a significant negative correlation between CEO social capital and the implied cost of capital. However, in the unique context of family businesses, CEO social capital serves as an informal mechanism that fosters the development of strong social connections between the family system and external stakeholders. This dynamic may be influenced by various factors, including the family governance structure, industry-specific characteristics, and the macroeconomic environment. Therefore, it is essential to conduct a comprehensive examination of the specific conditions under which CEO social capital can positively impact equity financing in family businesses.

#### 5.2.1 Family governance situations.

**(1) Family-formed methods:** Chinese family businesses primarily develop into either entrepreneurial or non-entrepreneurial types through direct or indirect pathways. Entrepreneurial family businesses are established by individuals or families and have remained family-controlled since their inception. Such families typically possess a strong sense of identity with the enterprise, prioritize the long-term goal of “sustaining growth,” and demonstrate both motivation and capability to overcome financing challenges through the CEO’s knowledge and social connections. Consequently, entrepreneurial families are also more inclined to introduce professional non-family managers. The entrepreneurial experience and personal prestige of the CEO enhance the role of social capital, making it more powerful and positive.

In contrast, non-entrepreneurial families achieve control through methods such as share restructuring, equity transfer, backdoor listings, and other maneuvers. These families generally exhibit a lower level of identification with the enterprise, making it challenging for CEO social capital to effectively mitigate expectations of market uncertainty. To investigate this dynamic, this section utilizes the `familial approach’ as a grouping variable to validate the previous analysis.

As shown in Columns (1) and (2) of Table 11, the SC coefficient for entrepreneurial families is -0.2955 (p <  0.01), while for non-entrepreneurial families, it is -0.0075 (p >  0.1). This indicates that CEO social capital significantly reduces the implied cost of capital only within entrepreneurial family enterprises.

**(2) Family control:** The high concentration of control within family members in family businesses can pose obstacles to their economic growth and value creation. The degree of family control significantly affects the ease and cost of obtaining external resources. When control is excessively rigid, family businesses often prioritize the rights and influence of family members, sometimes even at the expense of economic interests. This approach restricts the CEO’s ability to leverage their social capital to make a positive impact on equity financing beyond family boundaries. Conversely, when family control is more flexible, there is less incentive for collusion or self-serving behavior. This environment fosters a more open approach to hiring professional managers, attracting skilled talent, and courting external investors. It also creates opportunities for the CEO to leverage their social networks to broaden financing options and reduce capital costs. Overall, lower family control facilitates the beneficial role of CEO social capital. As Table 11 illustrates, the SC coefficient in column (4) is notably high and statistically significant, indicating a strong negative correlation. In contrast, the coefficient in column (3) is minimal and not statistically significant. Thus, when family control is relatively low, the CEO’s social capital may decrease the implicit cost of capital.

**(3) Intergenerational inheritance:** In China, family businesses are currently experiencing a peak in intergenerational succession. During this phase, for those enterprises that have not yet undergone succession, the founding generation typically enjoys a high degree of personal authority and management capability, making it easier to secure the trust and recognition of stakeholders. However, for businesses that have already completed the intergenerational succession, second-generation successors may exhibit tendencies of complacency and free-riding, and internal family involvement often exacerbates conflicts. In such cases, the CEO’s embedded social capital may increasingly exert a negative impact on multiple relationship conflicts, potentially leading external investors to raise risk premiums in response to legitimacy and operational risks, thereby significantly increasing the cost of equity capital.

To verify this conjecture, a group regression analysis was conducted based on whether the second generation was involved. As shown in column (5) of Table 11, the SC coefficient for family enterprises with intergenerational inheritance is 0.0621 (*p >  0.1*). Although this finding is not statistically significant, it indicates that CEO social capital tends to increase the cost of capital. In contrast, in column (6) for family businesses that have not yet undergone intergenerational succession, the SC coefficient is -0.4299 (*p <  0.01*). This suggests that CEO social capital can reduce the implied cost of capital only in family enterprises that have not experienced intergenerational inheritance.

#### 5.2.2 Industry characteristics.

**(1) Market competition:** As market competition intensifies, corporate profit margins are further compressed, making it increasingly difficult to rely solely on internal family resources to manage the risks and uncertainties associated with fierce market competition. In highly competitive industries, the costs and challenges of obtaining external financing increase, prompting investors to demand greater bargaining power and risk compensation. Consequently, companies must increasingly depend on the CEO’s knowledge, experience, and social network to provide risk-taking capabilities and credibility assurance. In such a market environment, CEO social capital becomes crucial for gaining stakeholder recognition and mitigating the pricing impacts of information asymmetry.

Drawing on Ramaswamy (2001) [39], this study employs the Herfindahl-Hirschman Index (HHI) to assess market competition, which calculates market share based on the primary business revenues of individual firms. A higher Herfindahl Index indicates lower industry competition. Building on this, this research designates HHI as a dummy variable: if HHI exceeds the median, it signifies a group with lower industry competition; otherwise, it denotes a group with higher competition. The group regression results are displayed in Table 12. In column (1), the SC coefficient is -0.4602 (p <  0.01); in column (2), the SC coefficient is -0.2510 (p <  0.1). These findings suggest that when industry competition is relatively high, CEO social capital has a more pronounced effect on reducing implicit capital costs.

**(2) High-tech industry:** High-tech enterprises require significant R&D funding and operate within a high-risk, high-reward framework, which provides them with substantial bargaining power for acquiring low-cost funding. When market investors are cautious, CEOs leverage their personal reputation and social connections to underscore their companies’ investment value and growth potential. Consequently, CEO social capital plays a pivotal role in the high-tech sector, significantly lowering the equity financing costs for family firms.

This study defines high-tech industries to encompass sectors such as medicine, aviation, electronics, communications, computers, chemistry, and scientific research. The regression analysis in columns (3) and (4) of Table 12 reveals key findings. In the high-tech sample, the SC coefficient is -0.5873 (p <  0.01), indicating a strong negative correlation. In contrast, the SC coefficient for non-high-tech industries is smaller at -0.2137, with a p-value greater than 0.1. This underscores the critical role of CEO social capital specifically within high-tech enterprises.

**Table 11:** Analysis of family characteristics.

Variables	The formation of family business		Family control		Intergenerational inheritance	
	Entrepreneurial	non-entrepreneurial	high	low	happened	hasn’t happened
	(1) ICC	(2) ICC	(3) ICC	(4) ICC	(5) ICC	(6) ICC
SC	-0.2955 ^***^ (-2.6576)	-0.0075 (-0.0290)	-0.0398 (-0.2510)	-0.4109 ^***^ (-2.9964)	0.0621 (0.2229)	-0.4299 ^***^ (-3.8381)
_cons	20.9486 ^***^ (6.6493)	46.0428 ^***^ (5.7669)	25.9299 ^***^ (5.3980)	18.3588 ^***^ (3.5482)	20.3402 ^***^ (3.3934)	22.9681 ^***^ (6.8926)
Control	Yes	Yes	Yes	Yes	Yes	Yes
time fixed effects	Yes	Yes	Yes	Yes	Yes	Yes
firm fixed effects	Yes	Yes	Yes	Yes	Yes	Yes
N	10286	1526	5907	5905	2061	9751
adj. R ^2^	0.2765	0.4257	0.2524	0.3252	0.3137	0.2900

#### 5.2.3 External macro environment.

**(1) Economic policy uncertainty:** The uncertainty economic policies exacerbates the ambiguity regarding the future operational prospects of enterprises, leading to fluctuations in their internal profits and cash flows. Consequently, market investors become increasingly cautious about future projections, driving up the market risk premium and, in turn, increasing the cost of equity capital. During periods of heightened economic uncertainty, family enterprises face growing demands for risk premium compensation and higher investment income expectations, which complicates their efforts to maintain stable capital costs. In this context, CEO social capital can play a crucial role in reducing the implied capital costs for family enterprises. Specifically, CEO social capital acts as an informal mechanism that offers investors supplementary information, helping to mitigate the investment risks associated with environmental uncertainty.

Utilizing the economic policy uncertainty index developed by Baker (2016) [40], this paper annualizes the directly obtained monthly data. The median was used as the critical value to distinguish between the relatively high group (UC =  1) and the low group (UC =  0). According to columns (1) and (2) of Table 13, in the relatively high group, the SC coefficient is -0.3125 (*p <  0.01*); in contrast, in the relatively low group, the SC coefficient decreases to -0.2299 (*p >  0.1*). These results indicate that when economic policy uncertainty is high, CEOs with robust social capital leverage their connections (e.g., professional networks and relational trust) to secure favorable financing terms, thereby significantly lowering the implied cost of capital (ICC) for family enterprises.

**Table 12:** Analysis of industry characteristics.

Variables	market competition		High-tech industry	
	High	Low	High-tech	Non-high-tech
	(1) ICC	(2) ICC	(3) ICC	(4) ICC
SC	-0.4602 ^***^ (-3.2013)	-0.2510 (-1.7877)	-0.5873 ^***^ (-4.1007)	-0.2137 (-1.4825)
_cons	21.2888 ^***^ (4.5416)	25.0553 ^***^ (6.1351)	26.2685 ^***^ (7.2007)	22.2938 ^***^ (4.7927)
control variables	Yes	Yes	Yes	Yes
time fixed effects	Yes	Yes	Yes	Yes
firm fixed effects	Yes	Yes	Yes	Yes
N	5939	5873	6071	5741
adj. R ^2^	0.2879	0.2919	0.3067	0.2777

**Table 13:** Analysis of the external macro environment.

Variable	Economic policy uncertainty		Investor legal protection	
	High	Low	Weak	High
	(1) ICC	(2) ICC	(3) ICC	(4) ICC
SC	-0.3125 ^***^ (-2.7174)	-0.2299 (-1.4200)	-0.2338 ^*^ (-1.8509)	-0.2266 (-1.4040)
Constant term	49.1313 ^***^ (9.0761)	8.7961 ^**^ (2.0341)	39.6903 ^***^ (10.0014)	17.8820 ^***^ (3.7028)
control variables	Yes	Yes	Yes	Yes
time fixed effects	Yes	Yes	Yes	Yes
firm fixed effects	Yes	Yes	Yes	Yes
N	5613	6199	5982	5830
adj. R ^2^	0.4341	0.2393	0.3213	0.2768

**(2) Investor legal protection:** When the legal protection environment for investors is stronger, the default cost for market participants tends to be lower. With improvements in information transparency between enterprises and investors, the role of CEO social capital diminishes. However, when investor legal protection is weak, investors rely more on informal social relations and trust to obtain supplementary information. In this case, CEO social capital mitigates investor risk compensation by functioning as an ``implicit” contract execution mechanism.

The legal environment index scores for various provinces and cities are used to measure investor legal protection, distinguishing between relatively strong and relatively weak groups based on the median. According to columns (3) and (4) of Table 13, in areas with relatively weak legal protection for investors, the SC coefficient is -0.2338 (p <  0.01). At the level of 1%, CEO social capital can significantly reduce the implied capital cost of family businesses. However, in areas with better legal protection, the SC coefficient decreased to -0.2266 (p >  0.1), and was no longer significant. This indicates that in regions with weak legal protection for investors, CEO social capital, as an informal system, has a more positive complementary effect and can significantly lower corporate capital costs.

## 6 Conclusion

This study explores the interplay between CEO social capital and corporate implicit capital costs, utilizing a dataset from family-listed firms trading on China’s A-share market from 2008 to 2021. The research reveals that CEO social capital significantly mitigates implicit capital costs for family firms, though this effect is partially offset by the CEO’s family-related background. Family CEOs are found to be less effective in reducing these costs compared to their non-family counterparts. The analysis suggests that CEO social capital achieves cost reductions by diminishing business risks and enhancing the transparency of information. Within the sphere of family governance, CEO social capital is particularly influential in lowering implicit capital costs for firms with reduced control and those that have not completed a generational succession. The study also finds that the impact of CEO social capital on cost reduction is more pronounced in industries with high competition and technological advancement. Furthermore, in environments characterized by significant economic policy uncertainty and weak investor legal protection, CEO social capital serves as a crucial mechanism for cost reduction within informal systems.

The conclusions of this paper offer important implications and recommendations for the practice of family businesses:

**Leverage CEO social capital:** Family businesses should strive to overcome the limitations imposed by their family systems and fully utilize the social capital of their CEOs. By facilitating the transfer of entrepreneurs’ social capital to the organizational level, these businesses can achieve synergies between internal and external resources, ultimately lowering the equity capital costs of family enterprises.

**Evaluate family control dynamics:** The findings indicate that social capital is not significantly impactful for family CEOs, and that family control and intergenerational inheritance do not enhance the positive effects of CEO social capital. This suggests that centralized family control and excessive protection of family interests may hinder the ability of CEO social capital to transcend familial constraints and fulfill its intended positive role.

**Integrate professional management:** During critical phases of transformation and intergenerational succession, Chinese family enterprises should consider bringing in professional managers or non-family capital. By strengthening the family governance structure and leveraging the complementary governance effects of diverse property rights, social capital can be maximized to effectively address the financing challenges that family enterprises face.

**Enhance competitiveness in high-tech industries:** In industries characterized by fierce product market competition and technological advancements, it is crucial to fully harness CEO social capital. This can help enhance collaborative governance through informal systems, improving both market competitiveness and the legitimacy of equity financing.

**Mitigate financing challenges:** Social capital can effectively mitigate the adverse selection problems arising from income uncertainty and investment risks, thereby reducing equity risk premiums and transaction costs associated with financing. This can alleviate the challenges of expensive and difficult financing that many family businesses encounter, thus promoting the high-quality development of China’s capital market and private economy. However, it is essential to ensure that the utilization of social capital operates within legal and ethical frameworks, safeguarding market fairness and protecting investor interests.
